# Coronary Artery and Peripheral Vascular Disease in a Patient with Poorly Differentiated Thyroid Cancer Treated with the Tyrosine Kinase Inhibitor Lenvatinib

**DOI:** 10.1155/2023/8841696

**Published:** 2023-10-31

**Authors:** Vineeth Sukrithan, Lisa Kim, Jennifer A. Sipos, Ashima Goyal, Ye Zhou, Daniel Addison, Manisha Shah, Bhavana Konda, Ajay Vallakati

**Affiliations:** ^1^Department of Internal Medicine, Division of Medical Oncology, The Ohio State University Wexner Medical Center, Columbus, OH, USA; ^2^Department of Internal Medicine, Division of Cardiology, The Ohio State University Wexner Medical Center, Columbus, OH, USA; ^3^Department of Internal Medicine, Division of Endocrinology, The Ohio State University Wexner Medical Center, Columbus, OH, USA

## Abstract

A subset of patients with differentiated thyroid carcinoma develop radioiodine refractory (RAIR) incurable disease, which typically has a poor prognosis. The multitargeted tyrosine kinase inhibitor lenvatinib has demonstrated significant improvements in progression-free survival in RAIR thyroid cancers compared to placebos. However, in the phase III SELECT trial of the drug in thyroid cancer, 5.4% of patients on lenvatinib experienced arterial thromboembolic events, with 2.7% experiencing severe grade ≥3 toxicities associated with arterial vascular events. This case study reports a patient with metastatic poorly differentiated follicular thyroid cancer who developed significant obstructive coronary artery disease following initiation of lenvatinib treatment, despite no predisposing cardiovascular risk factors apart from a remote smoking history. The possibility of developing coronary or peripheral artery disease should be considered in patients who are on targeted therapies, such as lenvatinib, even in the absence of traditional cardiovascular risk factors. In addition, baseline cardiac risk assessment and early treatment should be pursued to minimize interruptions to potentially lifesaving cancer therapy.

## 1. Introduction

Differentiated thyroid carcinoma, including papillary and follicular subtypes, comprises 90% of all thyroid cancers. Some patients with differentiated thyroid carcinoma develop radioiodine refractory (RAIR) incurable disease, which is associated with a poor prognosis [1]. In 2015, the US Food and Drug Administration (FDA) approved the multitargeted tyrosine kinase inhibitor lenvatinib following the results of the randomized, phase III SELECT trial, which demonstrated significant improvements in progression-free survival in RAIR thyroid cancers compared to placebos. Notably, 75.9% of patients experienced significant adverse events with toxicities classified as grade 3 or higher in this trial. Here, we present a case of coronary artery and peripheral vascular disease in a patient with thyroid cancer treated with lenvatinib.

## 2. Case Presentation

A male patient in his early 70 s with a history of benign prostatic hyperplasia, obstructive sleep apnea, and former tobacco use was diagnosed with metastatic poorly differentiated follicular thyroid cancer in 2014. He underwent a total thyroidectomy in 2014, followed by radioactive iodine therapy and external beam radiotherapy in 2015. Due to disease progression, he was enrolled in a randomized, double-blind, phase II trial of lenvatinib in patients with RAIR differentiated thyroid carcinoma in May 2018. He then developed secondary hypertension and initially needed carvedilol and lisinopril, which were later discontinued after his blood pressure normalized.

Fourteen months after starting lenvatinib, he presented to the emergency department with substernal chest pain. Symptoms included typical features including exertional angina with diaphoresis and nausea, which resolved within 20 minutes. Atypical features included association with meals. He had a prior 20 pack-year smoking history and quit in 1978. Otherwise, the patient denied traditional cardiovascular risk factors including hyperlipidemia, hypertension, diabetes, obesity, physical inactivity, or family history of coronary artery disease. An electrocardiogram (ECG) on arrival revealed sinus rhythm without acute ischemic changes. Laboratory tests showed mild elevation of troponin (0.25 ng/mL, normal < 0.045 ng/mL). A transthoracic echocardiogram was not performed as a recent test demonstrated normal left ventricular systolic function. The patient was discharged with no further cardiovascular testing. At the time of discharge, he was started on aspirin, atorvastatin, and carvedilol.

**Figure 1:**
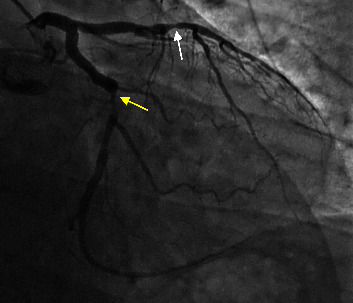
Obstructive coronary artery disease demonstrated on the coronary angiogram. Obstructive coronary artery disease was seen after 18 months of lenvatinib therapy. The coronary angiogram demonstrated mid-LAD with ∼90% stenosis (white arrow) (treated by placing two DES), moderate distal LAD disease, mid-LCx with ∼90% stenosis (yellow arrow) (treated by placing one DES), and distal left circumflex disease. LAD = left anterior descending artery; LCx = left circumflex artery; DES = drug-eluting stent.

He subsequently presented to the cardiology clinic with recurrent chest pain. A computerized tomography (CT) coronary angiogram was performed, which demonstrated severe stenosis with noncalcified plaque creating >70% stenosis of the midleft anterior descending (LAD) and distal left circumflex (LCx) arteries. He underwent cardiac catheterization, which confirmed significant obstructive coronary artery disease with 90% stenosis of mid-LAD and 90% stenosis of the mid-LCx arteries. Percutaneous coronary intervention was successfully performed with placement of two drug-eluting stents to mid-LAD and one to the mid-LCx arteries (Figure 1). In addition to dual antiplatelet therapy with aspirin and clopidogrel, the patient was placed on atorvastatin and metoprolol succinate. Further optimization of medical therapy was limited by hypotension and myalgias. Lenvatinib therapy was interrupted for one month given the lack of alternative explanations for his coronary artery disease.

**Figure 2:**
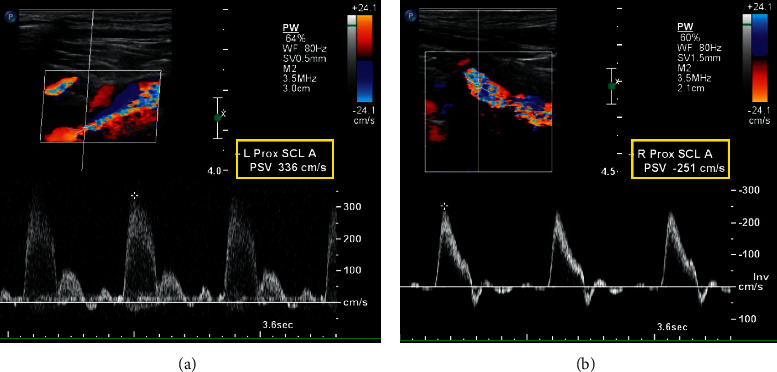
Bilateral subclavian artery stenosis demonstrated on carotid duplex ultrasound. Peak systolic velocity of the (a) proximal subclavian artery measured 336 cm/s and (b) proximal subclavian artery measured 251 cm/s.

One month later, restaging scans demonstrated an incidental finding of new severe bilateral subclavian artery stenosis located at the thoracic outlet, which was not present at the start of treatment (Figure 2). The vascular surgery team recommended conservative management with aspirin and clopidogrel for asymptomatic peripheral vascular disease. Due to further disease progression while off lenvatinib, the patient was removed from the clinical trial; however, lenvatinib was resumed at a reduced dose of 18 mg daily. He later developed new osseous metastasis requiring palliative radiation therapy, and a new left temporal meningioma treated with stereotactic radiosurgery. If further disease progression were to occur, the plan was to transition to cabozantinib based on data from a phase III trial [2].

## 3. Discussion

Improvements in progression-free survival must be carefully balanced with cardiotoxicities related to targeted therapies. The first case report of myocardial infarction associated with lenvatinib occurred after 39 months of treatment, resulting in stent placement, dual-chamber pacemaker implantation, and lenvatinib discontinuation [3]. Here, we describe a patient who developed obstructive coronary artery disease within 14 months of lenvatinib initiation, requiring coronary revascularization. This patient lacked predisposing cardiovascular risk factors apart from a remote smoking history. Baseline ECGs and the echocardiogram prior to lenvatinib were not suggestive of prior myocardial infarction. Similarly, baseline CT of the neck with contrast at the start of treatment did not reveal carotid or subclavian artery stenosis. Restaging scans after 14 months of treatment with lenvatinib revealed severe stenosis of the bilateral subclavian and carotid arteries. Hypertension after initiation of lenvatinib followed by normalization of blood pressure suggests recent development of severe bilateral subclavian artery stenosis. External beam radiotherapy to the neck probably contributed to the development of subclavian artery stenosis, but it is unlikely that radiation therapy resulted in obstructive coronary artery disease. In the absence of alternative explanations, lenvatinib-associated atherosclerosis of the coronary and subclavian arteries was considered to be the most likely etiology.

Treatment with multikinase inhibitors such as lenvatinib increases the risk of acute coronary syndromes and arterial thromboembolic events including cerebrovascular events, transient ischemic attacks, and myocardial infarction [1, 4]. In the phase III trial of lenvatinib in thyroid cancer (SELECT), 5.4% of patients on lenvatinib experienced arterial thromboembolic events, with 2.7% experiencing severe grade ≥3 toxicities [1, 5]. Pathophysiology is related to its antiangiogenic properties through vascular endothelial growth factor (VEGF) inhibition, which causes alterations in vascular hemostasis and induces endothelial injury, arterial thrombosis, and a procoagulant state [5–7]. This increases the risk of developing coronary artery disease and acute coronary syndromes due to coronary vasospasm, thrombosis, and progression of atherosclerotic plaques [4, 7]. It is worth noting that there is conflicting evidence of the role of VEGF-A in the development of atherosclerosis. Some preclinical studies paradoxically implicate VEGF-A in the promotion of atherogenesis by preventing the repair of endothelial lesions and promoting monocyte adhesion, migration, and activation leading to endothelial dysfunction [8, 9]. However, the totality of clinical experience from the use of anti-VEGF TKIs seems to indicate a proatherosclerotic effect [10].

Identifying pre-existing cardiovascular disease is of paramount importance in those undergoing treatment with novel targeted therapies. Careful cardiovascular surveillance is required to minimize the likelihood of adverse cardiac events and interruption of potentially lifesaving therapies [7]. Conventional cardiovascular risk tools may underestimate the risk in patients receiving vasotoxic agents [11]. We suggest that clinicians consider vasoactive therapy as a potential cardiovascular risk factor. According to the Society for Cardiovascular Angiography and Interventions (SCAI) expert consensus statement, patients receiving vasoactive cancer therapies should undergo baseline cardiac evaluation including assessment of atherosclerotic disease risk. For further risk stratification, coronary CT angiography or noninvasive testing is recommended for asymptomatic patients every 5 years during or after treatment [12]. In symptomatic patients, coronary angiography should be pursued. This case demonstrates that lenvatinib may increase the risk for the development of coronary and peripheral arterial disease.

## 4. Conclusion

The possibility of developing coronary or peripheral artery disease should be considered in patients who are on targeted therapies, such as lenvatinib, even in the absence of traditional cardiovascular risk factors. In addition, baseline cardiac risk assessment and early treatment should be pursued to minimize interruptions to potentially lifesaving cancer therapy. Further investigation is needed to elucidate the underlying mechanism causing atherosclerosis in patients receiving lenvatinib. Ultimately, the benefits of novel targeted therapies must be balanced with the risks of emerging drug-related cardiotoxicities.
